# Modeling scatter through sides of island blocks used for intensity‐modulated bolus electron conformal therapy

**DOI:** 10.1002/acm2.13889

**Published:** 2023-01-07

**Authors:** Joseph G. Scotto, Garrett M. Pitcher, Robert L. Carver, Kevin J. Erhart, Andrew S. McGuffey, Kenneth R. Hogstrom

**Affiliations:** ^1^ Department of Physics and Astronomy Louisiana State University Baton Rouge Louisiana USA; ^2^ Mary Bird Perkins Cancer Center Baton Rouge Louisiana USA; ^3^ decimal, LLC Sanford Florida USA

**Keywords:** bolus electron conformal therapy, electron scatter, electron therapy, intensity modulation

## Abstract

**Purpose:**

Passive Radiotherapy Intensity Modulators for Electrons (PRIME) devices are comprised of cylindrical tungsten island blocks imbedded in a machinable foam slab within the patient's cutout. Intensity‐modulated bolus electron conformal therapy (IM‐BECT) uses PRIME devices to reduce dose heterogeneity caused by the irregular bolus surface. Heretofore, IM‐BECT dose calculations used the pencil beam redefinition algorithm (PBRA) assuming perfect collimation. This study investigates modeling electron scatter into and out the sides of island blocks.

**Methods:**

Dose distributions were measured in a water phantom at 7, 13, and 20 MeV for devices having nominal intensity reduction factors of 1.000 (foam only), 0.937, 0.812, and 0.688, corresponding to nominal island block diameters (*d_nom_*) of 0.158, 0.273, and 0.352 cm, respectively. Pencil beam theory derived an effective diameter (*d_IS_*) to account for in‐scattered electrons as a function of *d_nom_* and beam energy (*E_p,0_*). However, for out‐scattered electrons, an effective diameter (*d_mod_*) was estimated by best fitting measured data.

**Results:**

In the modulated region (under island blocks, depth < R_90_), modified PBRA‐calculated dose distributions showed 2%/2 mm passing rates for *d_nom_* = 0.158, 0.273, and 0.352 cm of (100%, 100%, 100%) at 7 MeV, (100%, 100%, 93.5%) at 13 MeV, and (99.8%, 85.4%, and 71.5%) at 20 MeV. The largest dose differences (≤ 6%) occurred at the highest energy (20 MeV), largest *d_nom_*, shallowest depths (≤ 2 cm), and on central axis.

**Conclusions:**

An equation for modeling island block scatter, *d_mod_* (*d_nom_*, *E_p,0_*), has been developed for use in the PBRA, insignificantly impacting calculation time. Although inaccuracy sometimes exceeded our 2%/2 mm criteria, it could be clinically acceptable, as superficial dose differences often fall inside the bolus. Also, patient PRIME devices are expected to have fewer large diameter island blocks than did test devices. Inaccuracies are attributed to out‐scattered electrons having energy spectra different than the primary beams.

## 1 INTRODUCTION

Bolus electron conformal therapy (BECT) is a common treatment modality that uses a patient‐specific variable thickness bolus situated on the patient's surface to modulate the range of the electrons. In this technique, a single electron beam is used to treat the planning target volume (PTV) with a beam energy sufficient to adequately cover the deeper portions of the PTV. The range of the electrons are modulated laterally using a bolus of variable thickness designed to conform the 90% isodose surface to the distal PTV surface,^1^, ^2^ where 100% equals given dose, which is the central‐axis dose maximum to a water phantom at the treatment SSD for the rectangle of least area that circumscribes the treatment field.

BECT has the benefit of providing adequate dose coverage to the PTV, while reducing dose to distal critical structures and normal tissues and ensuring low whole‐body dose. BECT has been shown useful for treatment of post‐mastectomy chest wall^3^, ^4^, ^5^, ^6^, ^7^; shallow sarcomas^3^, ^4^, ^8^; ear, temple,^9^ parotid, and buccal mucosa^4^, ^10^; nose^11^; eye canthi^12^, ^13^; and extremities.^8^ Currently, BECT is commercially available from two companies (.decimal, LLC, Sanford, FL and Adaptiiv Medical Technologies, Inc., Halifax, NS, Canada) that provide software based on bolus design algorithms,^8^, ^14^ integrate that software with the customer's treatment planning system, and allow for bolus fabrication through either milling of machinable wax^14^, ^15^ or 3D printing.^7^, ^8^, ^16^

Although BECT improves dose conformity, gradients in the upstream bolus surface can prevent PTV dose homogeneity (ideally 90–100%), which Kudchadker et al.^2^ showed as severe as 30% (90–120%). However, that study demonstrated that introducing intensity modulation followed by slightly modifying the bolus shape can restore PTV dose homogeneity to be close to its typically ideal 90–100%, while maintaining conformity of the distal 90% dose surface to the PTV.^4^

In 2017, Hogstrom et al. introduced Passive Radiotherapy Intensity Modulation for Electrons (PRIME) devices as a practical, novel technology for delivering electron intensity modulation.^17^ This patented technology^18^ describes PRIME devices, simply known as intensity modulators, which utilize cylindrical tungsten island blocks of varying diameters in a hexagonal pattern within the electron field to passively modulate the beam intensity. The 0.6 cm long island blocks are imbedded in a low‐density machinable foam slab situated within a patient‐specific collimating insert (cutout). They are oriented to align with the divergence of the beam such that their axes project back to the nominal source position (100 cm upstream of isocenter).^9^, ^17^, ^19^, ^20^ Incident electrons are absorbed by the island blocks so that the underlying fluence in the patient is reduced locally by approximately the fraction of the cross‐sectional area of the field covered by the overlying nearby island blocks. This is possible because lateral scattering of the electrons, primarily due to air upstream of the intensity modulator and the machinable foam slab in which it is embedded, restores locally the downstream fluence uniformity, but to a reduced value. The relative local planar fluence (intensity) is reduced approximately to the nominal intensity reduction factor (IRF*_nom_*), which is the unblocked fraction of the beam cross section. For island blocks of physical (nominal) diameter 𝑑*_nom_* arranged on a hexagonal grid with packing radius 𝑟, IRF*_nom_* is geometrically computed to be^17^

(1) $$\mathrm{IR}F_{nom}\left(r,d\right)=1-\left(\frac{\pi }{2\sqrt{3}}\right){\left(\frac{d_{nom}}{r}\right)}^{2}.$$

The application of PRIME‐based intensity modulation to BECT (IM‐BECT) has been shown to improve dose homogeneity in the PTV for treatment of post‐mastectomy chest wall and temple.^9^, ^21^ However, clinical implementation of this treatment modality requires accurate dose calculation. BECT, as marketed by .decimal, LLC (Sanford, FL), uses the pencil beam redefinition algorithm (PBRA)^22^, ^23^, ^24^, ^25^ for dose calculation, whose accuracy has been well documented in anthropomorphic phantoms for BECT.^26^, ^27^ The PBRA calculates dose as the sum of two components, the primary electrons, *D_e_*, and the background x‐ray dose, *D_x_*_,_

(2) $$D\left(x,y,z\right)=D_{e}\left(x,y,z\right)+D_{x}\left(x,y,z\right).$$

Modifications were made to the primary electron component to account for the presence of the PRIME devices. First, for primary electrons not incident on the upstream surface of the island blocks, electron scatter and energy loss in the PRIME's 1.27 cm thick machinable foam (ρ = 0.096 g cm^−3^) in which the blocks are imbedded was incorporated by increasing 𝜎_𝜃𝑥_ by 50%, reducing *R*_90_ range by 0.1 cm, and reducing *E_p,0_* by 0.2 MeV.^9^, ^20^ Second, the model approximated the island blocks as perfect collimators, that is, assumed that no electrons scattered into or out of the sides of the island blocks. Therefore, all electrons incident on the upstream surface of the island block were assumed to be absorbed and all others transmitted to the patient plane.^9^, ^17^, ^19^ To model perfect collimation, the planar fluence distribution for the *n^th^* energy bin resulting from a small square pencil beam (side = $s_{i}$) of area equal to the circular cross section of each *i^th^* island block was transported from the collimator plane to points (x,y,z) on the patient surface. Then, the planar fluence for each small square pencil beam was subtracted from the primary electron fluence of the *n^th^* energy bin resulting from the open beam (without island blocks), that is,

(3) $$\phi_{PRIME}^{n}\;\left(x,y,z\right)=\phi_{open}^{n}\;\left(x,y,z\right)-\sum_{i\;=\;1}^{N}\phi_{IB,i}^{n}\left(x,y,z,\;x_{i},y_{i},s_{i}\right).$$

where $\phi_{\mathrm{open}}^{n}$ is the open field planar fluence (intensity) distribution in the *n^th^* energy bin at position (*x,y,z*) on the patient surface, $\phi_{IB,i}^{n}$ is the planar fluence distribution in the *n^th^* energy bin from the *i^th^* pencil beam simulating the island block located at (*x_i_,y_i_*) in the PRIME device, and $\phi_{PRIME}^{n}$ is the planar fluence distribution of the *n^th^* energy bin after incorporating the island blocks into the calculation.

**FIGURE 1:**
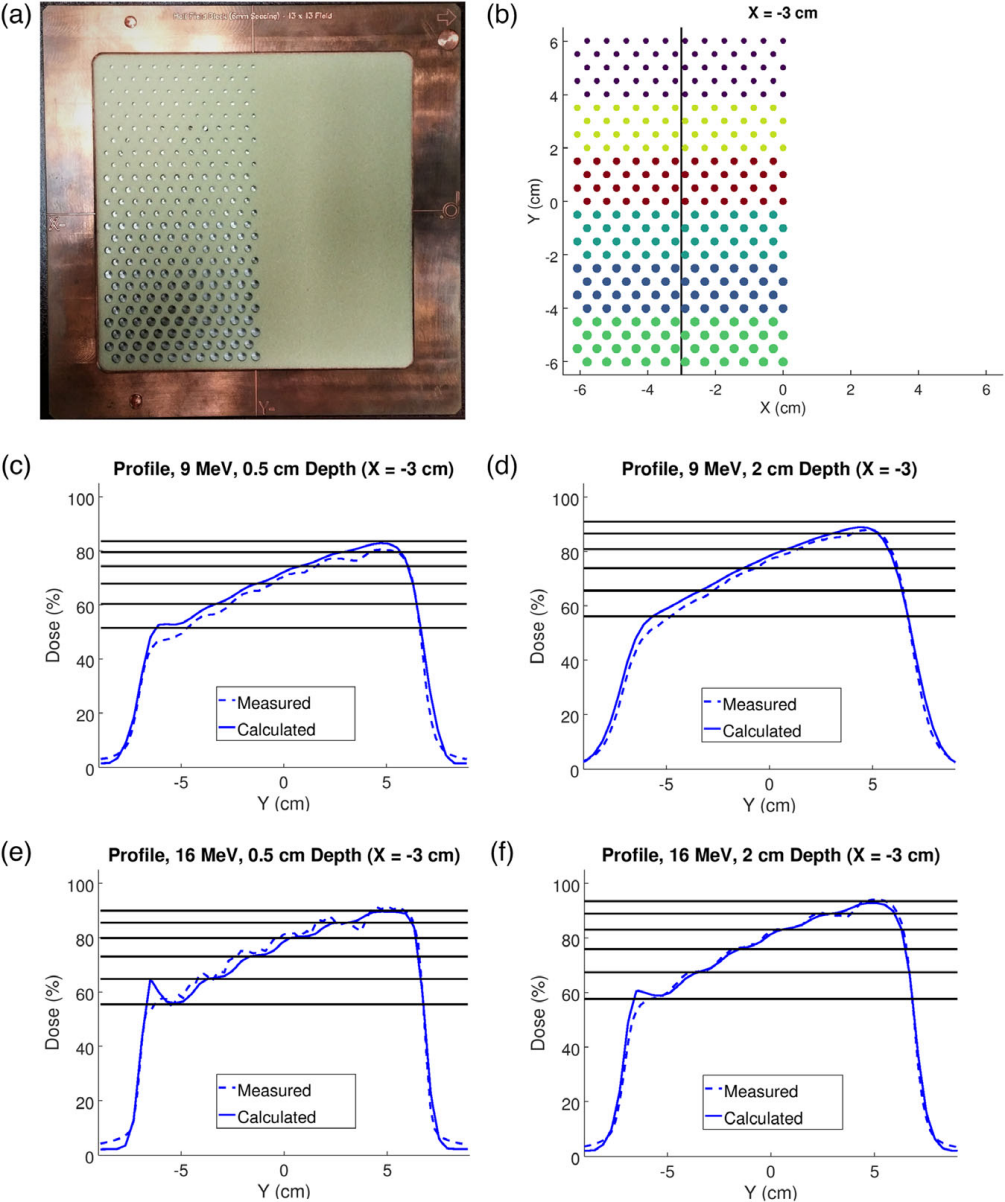
Dose measurements in water under test PRIME device for 9 and 16 MeV beams. (a) PRIME device fabricated by .decimal features half (x < 0) of a 13×13 cm^2^ field containing tungsten island blocks imbedded in a low‐density machinable foam. (b) Schematic of PRIME device shows different colored island block diameters change every four rows with island blocks located on a hexagonal grid (0.579 cm packing radius), resulting in IRFs increasing in the +y direction from ≈60% to 95%. The line at x = −3 cm demarcates the measurement plane containing the lateral dose profiles plotted in (c‐f). These plots compare measured (dashed) and PBRA‐calculated (solid) dose profiles for 9 and 16 MeV beams acquired at 0.5 and 2.0 cm depths in water; 100% = dose measured at R_100_, x = 3 cm, and y = 0 in the unmodulated half of beam (from Hilliard^20^)

However, measurements by Hilliard^20^ suggested that this “perfect collimation” approximation is not fully valid. Figure 1 compares Hilliard's measured lateral dose profiles with those calculated using the idealized “perfect collimator” implementation of the intensity modulated PBRA, as specified above. Figure 1(b) indicates the plane of scanned diode measurements obtained in a water tank below the PRIME device shown in Figure 1(a). The circles of various colors in Figure 1(b) indicate the modulator blocks of varying diameters ranging from 0.15  to 0.4 cm. Figures 1(c‐f) compare measured and calculated dose profiles in a water phantom at 0.5 and 2.0 cm depths underlying the device shown in Figure 1(a) for 9 and 16 MeV energy beams.

In‐scattering of electrons into the lateral surface of an island block reduces fluence, and hence dose, in the underlying water phantom by further removing electrons from the beam. Expected to be more prominent for lower energy electrons due to their greater scattering power, this effect is observed in Figures 1(c) and (d), which show the PBRA calculated dose greater than measured dose at depths of 0.5 and 2.0 cm in water for a 9 MeV beam.

Out‐scattered electrons, which enter the upstream or lateral surface of an island block and then scatter out of the side, increase underlying dose by contributing to the incident fluence. Out‐scattering is expected to be more prominent for higher energy electrons, due to their increased range increasing the likelihood that they escape the island block. This effect is observed in Figures 1(e) and (f), which compare measured dose with PBRA calculations at depths of 0.5 and 2.0 cm in water for a 16 MeV beam. At 0.5 cm depth, measurement exceeds PBRA‐calculated dose due to the out‐scattered electrons not being modeled. However, at 2.0 cm depth measured dose closely agrees with PBRA‐calculations. This is presumed due to many of the energy‐degraded, out‐scattered electrons failing to reach the deeper depth, and the effect of those that do being countered by in‐scattered electrons. Preliminary Monte Carlo investigations, which revealed the mean energy of out‐scattered electrons being reduced by as much as 44% for high‐energy beams, are discussed later.^28^

The current study reports on a model aimed at improving the accuracy of PBRA dose calculations by accounting for the effects of scatter into and out of the island blocks. In‐scattered electrons can be modeled using pencil beam theory, described below, which results in formulae for modifying the island block diameter as a function of beam energy and island block diameter. Out‐scattered electrons could not be modeled using pencil beam theory; therefore, measurements were made that allowed determination of an empirical formula for modifying the island block diameter that is a function of beam energy and island block diameter. Model parameters were least square fit to provide optimal agreement between measurement and PBRA calculation. The accuracy of the empirical model was assessed by comparing modified PBRA calculated with measured dose distributions.

## 2 THEORY OF IN‐SCATTERED ELECTRONS

Currently, the PBRA calculates the planar fluence distribution incident on the patient surface for each energy bin of each pencil beam using the initial energy spectrum^24^ at the PRIME device location and assuming perfect collimation, as described using Equation (3). The resulting planar fluence for the *n^th^* energy bin, $\phi_{pat,n}\left(x,y,z\right)$ at pencil beam location (x,y,z), can be modified for electrons scattered into and out of each island block by

(4) $$\phi_{pat,n}^{\prime }\left(x,y,z\right)=\phi_{pat,n}\left(x,y,z\right)-\Delta \phi_{n}^{IS}+\Delta \phi_{n}^{OS},$$

where $\Delta \phi_{n}^{IS}$ represents the decrease of planar fluence in the *n^th^* energy bin due to scattering into an island block (in‐scatter) and $\Delta \phi_{n}^{OS}$ is the increase in planar fluence in the *n^th^* energy bin due to scattering from an island block (out‐scatter). Because in‐scattered electrons are removed from the beam, it is assumed the incident energy distribution is insignificantly affected. For such case, a method is described next, which requires only modifying the island block diameters in Equation (3) to calculate together the first two terms of Equation (4). Computing $\Delta \phi_{n}^{OS}$ becomes more complicated if its calculation accounts for the scattered electrons energy spectrum being different from the initial energy spectrum. However, in the present work, we assumed the scattered energy spectrum was the same as the initial energy spectrum. This allowed $\Delta \phi_{n}^{OS}$ to be modeled by modifying each island block's diameter as a function of beam energy, *E_p,0_*, and physical island block diameter, 𝑑*_nom_*, just as done for $\Delta \phi_{n}^{IS}$.

The decrease in planar fluence due to in‐scatter can be estimated using pencil‐beam theory for a right, square parallelepiped of side *s*, whose cross‐sectional area (*s* ^2^) and thickness (*t*) equal those of a 0.6 cm long cylindrical island block of diameter 𝑑*_nom_*. Using pencil‐beam theory, it can be shown that the ratio of the number of electrons impinging the sides of the island block to the number incident on the upstream surface of the island block is given by

(5) $$\begin{array}{ccc} & & f\left(E_{p,0},d_{nom}\right)=1-\frac{2\sigma_{x}^{2}}{s^{2}}\\ & & {\left\{\frac{s}{\sqrt{2}\sigma_{x}}\mathrm{erf}\left(\frac{s}{\sqrt{2}\sigma_{x}}\right)+\frac{1}{\sqrt{\pi }}\left[\exp\left(-\frac{s^{2}}{2\sigma_{x}^{2}}\right)-1\right]\right\}}^{2}, \end{array}$$

where *E_p,0_* is the most probable incident energy of the beam, 𝑑*_nom_* is the physical island block diameter, and *s* is the side of a square of equal cross‐sectional area of the island block.^28^ $\sigma_{x}$, the RMS spread of the lateral distribution of a pencil beam traveling through the machinable foam for the thickness of the intensity modulator, is given by

(6) $$\sigma_{x}\left(t\right)={\left[\sigma_{\theta_{x,clinical}}^{2}+\frac{1}{6}{\left(\frac{T}{\rho }\right)}_{foam}\rho_{foam}t\right]}^{1/2}t,$$

where $\sigma_{\theta_{x},clinical}$ is the projected angular spread due to multiple Coulomb scattering in the dual scattering foils and air, as determined by measurement, ${\left(\frac{T}{\rho }\right)}_{foam}$ is the mass angular scattering power of the machinable foam at energy $E_{p,0}$, and $\rho_{foam}$ is the machinable foam density (0.096 g∙cm^−3^).

Hence, the fraction of electrons stopped in the island block as determined by Equation (1), that is, those incident on the proximal block surface, can be increased to also include electrons scattering into the side of an island block by

(7) $$\begin{array}{c} \phi_{IB,i}\;\left(x,y,z;x_{i},y_{i},d_{nom,i}\right)=\phi_{IB,perfect,i}\;\\ \left(x,y,z;x_{i},y_{i},d_{nom,i}\right)\cdot \left(1+f\left(E_{p,0},d_{nom,i}\right)\right) \end{array}$$

This is equivalent to changing the IRF in Equation (1) using

(8) $$\mathrm{IR}F_{IS}=\mathrm{IR}F_{nom}-f\left(E_{p,0},d_{nom,i}\right)\cdot \left(1-\mathrm{IR}F_{nom}\right),$$

and increasing the block diameter using

(9) $$d_{IS}\left(E_{p,0},d_{nom}\right)=d\sqrt{1+f\left(E_{p,0},d_{nom}\right)}.$$

The in‐scatter‐adjusted quantities, *f, d_IS_*, and IRF*_IS_*, for the three island block diameters and three clinical beam energies investigated in this study are listed in Table 1. For all energies, the additional fractional fluence lost, *f*, increases as the island block diameter decreases. In‐scattered electrons increase the cylindrical island block diameter 𝑑*_nom_* to an effective diameter *d_IS_* according to Equation (9), and results in Table 1 show this increase to be almost independent of block diameter. The magnitude of this increase from 𝑑*_nom_* to *d_IS_* decreases with energy, being approximately 0.032, 0.021, and 0.013 cm for the 7, 13, and 20 MeV beams, respectively. The in‐scattered electrons decrease values of IRF*_nom_* to IRF*_IS_* according to Equation (8), and results in Table 1 show the decrease to be greater as cylindrical block diameter increases and as beam energy decreases. These decreases range from 0.053 for a 0.352 cm diameter block at 7 MeV to 0.011 for a 0.158 cm diameter block at 20 MeV.

**TABLE 1:** Physical and in‐scatter‐adjusted island block diameters and IRFs for 7, 13, and 20 MeV beams

Energy (MeV)	*d_nom_* (cm)	IRF*_nom_*	*f*	*d_IS_* (cm)	IRF*_IS_*
7	0.158	0.937	0.420	0.188	0.911
	0.273	0.812	0.257	0.306	0.764
	0.352	0.688	0.202	0.386	0.625
13	0.158	0.937	0.268	0.178	0.920
	0.273	0.812	0.160	0.294	0.782
	0.352	0.688	0.125	0.373	0.649
20	0.158	0.937	0.172	0.171	0.926
	0.273	0.812	0.101	0.286	0.793
	0.352	0.688	0.079	0.366	0.663

The physical island block diameters (d_nom_) are listed with corresponding IRF_nom_ values calculated according to Equation (1) for a hexagonal grid spacing (r) of 0.6 cm. The f‐factors were calculated using Equation (5) and parameters found in Scotto^28^ for beam energies 7, 13, and 20 MeV. The resulting in‐scattered‐adjusted diameters (d_IS_) and respective IRF_IS_ values were calculated using Equations (9) and (8), respectively. Note that island block diameters (d_nom_ and d_IS_) and grid spacing (r) are specified in the plane of the collimating insert located 5 cm upstream of isocenter.

These equations, which provide a method for accounting for electrons scattered into the cylindrical island blocks, insignificantly increase computation time of PBRA calculations. Using this correction alone was found insufficiently accurate, even at the lower energies where in‐scatter predominates. However, it was not possible to derive such a set of equations to model out‐scattered electrons. This is due to electrons scattering from a island block having a wide range of energies and trajectories, due to varying exit depths from the tungsten cylinders. Therefore, the present study simply further modified the diameter of each cylindrical island block as a function of its diameter and beam energy to account for both in‐scattered and out‐scattered electrons. This solution assumes that out‐scatter does not change the electron energy spectrum, which is not the case, but does result in a solution that insignificantly increases time of PBRA dose calculation. An optimal island block diameter was determined as a function of beam energy and *d_nom_* by minimizing the differences between calculated and measured dose distributions. The major objective of this study was to determine a function for modifying island block diameters for inclusion in the PBRA algorithm for IM‐BECT dose calculations and to evaluate its accuracy.

## 3 METHODS

### 3.1 Measurements

**FIGURE 2:**
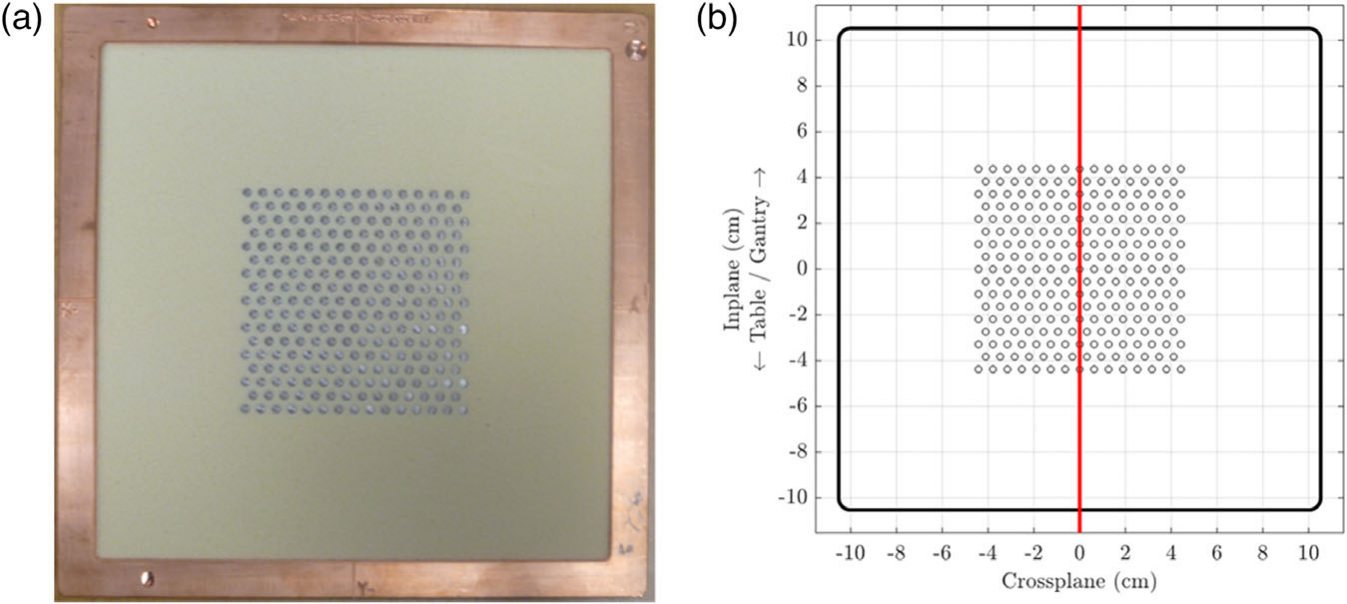
One of three test intensity modulators used to evaluate island block scatter. (a) Image shows the PRIME device comprised of 247 0.273 cm diameter island blocks placed on a 0.6 cm hexagonal grid. (b) The red line in its schematic drawing demarcates the measurement plane, which is in the accelerator's in‐plane dimension. The three test PRIME devices were all similar except for having 0.158, 0.273, and 0.352 cm island block diameters corresponding to IRF*_nom_* values of 0.937, 0.812, and 0.688 according to Equation (1)

Four PRIME devices were manufactured by .decimal, LLC (Sanford, FL) for accumulating measured data for comparison with PBRA‐calculated dose distributions. The devices consisted of 0.6 cm long cylindrical tungsten rods (island blocks) imbedded in a 1.27 cm thick slab of machinable foam (0.096 g cm^−3^), which was situated within a 21×21 cm^2^ (projected to isoceneter) copper insert. One device contained no island blocks, while the other three contained an 8.4×8.4 cm^2^ matrix of 247 island blocks placed on a hexagonal grid with 0.6 cm spacing (dimensions specified at applicator insert, 5 cm upstream of isocenter). The tungsten island blocks within the three devices had diameters (*d_nom_*) of 0.158, 0.273, and 0.352 cm, corresponding to IRF*_nom_* values, calculated using Equation (1), of 0.937, 0.812, and 0.688, respectively. Figure 2(a) shows the PRIME device fabricated with 0.273 cm diameter island blocks. Note that island blocks of intermediate nominal diameters of 0.223 and 0.315 cm, corresponding to nominal IRF*_nom_* values of 0.875 and 0.750, respectively, were not included in our measurements, though they are part of the set available from .decimal, LLC for treatment planning and delivery.^9^

Measurements were performed on the Elekta Infinity (Elekta, Inc., Atlanta, GA) at Mary Bird Perkins Cancer Center (MBPCC) using an IBA Blue Phantom Compact 2D scanning system with IBA myQA Accept control software (IBA Dosimetry, Bartlett, TN, USA). IBA CC13 ionization chambers (0.13 cm^3^ active volume, 0.6 cm inner diameter) were used as both field and reference detectors. All scanning was performed in the accelerator in‐plane direction.

Scans were performed with the 7, 13, and 20 MeV beams (*E_p,0_* values of 7.14, 13.12, and 20.47 MeV, respectively) at 100 SSD for each of the four intensity modulators (foam only; 0.158 , 0.273 , and 0.352 cm island block diameters). For all energy and intensity modulator combinations, ionization versus depth curves were collected along central axis from a depth of 12 cm to the surface. The depth of measurement (*z*) was physical depth less 0.5 times the radius of the diameter of the ion chamber's cylindrical air cavity. Ionization versus depth curves were converted to percent dose versus depth (PDD) curves using TG‐25 protocol^29^ using $\bar{E_{0}}$ values calculated from the foam‐only percent ionization versus depth curves. Dose output measurements (100 monitor units) acquired for each modulator were used to normalize the PDD of each modulator such that 100% equaled the given dose, i.e., *D_max_* of the foam only PDD measurement, for each beam energy.

In‐plane, off‐axis profiles from ‐12 to +12 cm, as indicated in Figure 2(b), were measured at multiple depths, that is, 0.5 to 3.5 cm in 0.5 cm increments for the 7 MeV beam and 0.5 cm to 6.5 and 9.5 cm for the 13 and 20 MeV beams, respectively, in 1.0 cm increments. All profiles of dose versus off‐axis distance (*y*) were centered, symmetrized, and normalized to *D_max_* of the foam only PDD for a given beam energy.

For the purpose of comparing with PBRA calculation, for each combination of beam energy and intensity modulator, PDD and off‐axis dose profiles were combined to create a measured, in‐plane 2D dose matrix and re‐gridded to have 0.1 cm resolution in both the lateral and depth dimensions. This was computed using

(10) $$D_{meas}\left(y,z\right)=PDD\left(z\right)\times OAR\left(y,z\right),$$

where *PDD(z)* is the PDD value at the measurement depth *z*, and *OAR(y,z)* is the linear interpolation between adjacent off‐axis (*y*) profiles.

### 3.2 PBRA dose calculations with nominal island block diameters

Dose calculations were performed in water using a developmental version of the PBRA code commissioned using MBPCC clinical data. Four calculations were performed for each of the 7, 13, and 20 MeV beams at 100 SSD in water for a 21×21 cm^2^ field, one containing only foam and three for the intensity modulators identical to the PRIME devices used for measurement. PRIME device specifications were read from a text file containing the total number of island blocks and their in‐plane and cross‐plane positions and diameters projected to isocenter (100 cm SAD).

To minimize error due to small beam characteristic fluctuations between measurement sessions, measured foam‐only PDDs (and respective *R_p_* and *E_p,0_* values) and off‐axis factors were used as PBRA model input for the calculations. The 0.1 cm *R*_90_ shift to account for the foam in the PBRA, as used by Hilliard,^20^ was excluded from the calculation, as the foam was present during measurement of the PDD used as input; however, the 50% increase in 𝜎_𝜃𝑥_ was retained. The quality of agreement of the commissioned PBRA‐calculated with measured dose distributions in water for the PRIME device with foam only at 7, 13 MeV, and 20 MeV was within approximately 1%, as previously reported by Boyd et al.^23^

### 3.3 Calculation evaluation and correction methods

Our nine sets (three beam energies × three island block diameters) of measured data revealed that PBRA calculations, as reported by Hilliard et al.,^9^ generally over‐predicted measured dose for low energy beams and under‐predicted dose for high‐energy beams, presumably due to neglecting scatter in and out the sides of the island blocks. To account for this scatter and improve calculation accuracy, empirical corrections were made to the amount of the electron fluence removed from the primary beam by the island blocks comprising the PRIME device. This was done by modifying each island block's diameter as a function of *d_nom_* and *E_p,0_*. Specifically, the modified diameter (*d_mod_*) was determined by minimizing least squares differences between measured and calculated data for each of the nine data sets individually. Each optimization was performed within the modulated region, defined as the region within ± 3.5 cm of central axis and from 0.5 cm to slightly beyond *R_90_* in depth, that is, 2.0, 4.5, and 6.5 cm depth for the 7, 13, and 20 MeV beams, respectively. For *N_y_* off‐axis calculation points at *N_z_* depths in the modulated region, the optimal value for *d_mod_* was determined such that

(11) $$\begin{array}{ccc} min & = & \sum_{i,j}^{N_{y},N_{z}}\left[D_{meas}\left(d_{nom},\;y_{i},z_{j}\right)\right.\\ & & -\;{\left.D_{PBRA}\left(d_{mod},y_{i},z_{j}\right)\right]}^{2}\;, \end{array}$$

where *D_meas_* and *D_PBRA_* are the measured and PBRA‐calculated dose values, respectively, at positions $\left(y_{i},z_{j}\right)$, which are the locations of each data point on the 0.1 cm square grid described earlier.

To extend the solution for *d_mod_* to intermediate island block diameters and beam energies, a least squares fit was applied to *d_mod_* values as a quadratic function of *d_nom_* and *E_p,0_*, that is,

(12) $$\begin{array}{c} d_{mod}\;\left(d_{nom},E_{p,0}\right)=A_{0}\;\;+\;A_{1}d_{nom}\;+\;A_{2}E_{p,0}\;\\ +\;A_{3}d_{nom}^{2}\;+\;A_{4}d_{nom}E_{p,0}+A_{5}E_{p,0}^{2} \end{array}$$

## 4 RESULTS

### 4.1 Modified island block diameters

**FIGURE 3:**
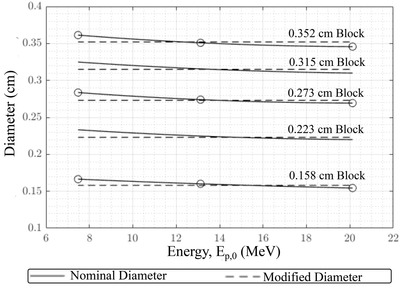
Nominal and modified island block diameters plotted versus beam energy. A PRIME device is a hexagonal grid (0.6 cm separation) of intensity modulators containing island blocks (0.6 cm long tungsten cylinders) of varying diameters from a specified set, whose data is plotted here. The circles indicate the block diameter values determined by a least square fit to measured data (*cf*., Equation 11) for the nine combinations of three island block diameters and three beam energies. The solid curves are fits to the circled points using the empirical quadratic curve in Equation (12). The dashed lines demarcate the nominal (physical) diameter of the three PRIME devices studied. The five nominal island block diameters of 0.158, 0.223, 0.273, 0.315, and 0.352 cm comprise a specified set having corresponding IRF*_nom_* values (*cf*., Equation 1) of 0.937, 0.875, 0.812, 0.750, and 0.688, respectively

Following the methodology above, modified island block diameters (*d_mod_*) versus beam energy (*E_p,0_*) for five nominal block diameters (*d_nom_*) are plotted in Figure 3. Results show that parameterization (solid lines) of *d_mod_* (Equation 12) closely fit the optimized values (open circles), which were obtained by least square fitting (Equation 11) the PBRA‐calculated to the measured dose distribution in the modulated region for each of the nine individual combinations of *d_nom_* and *E_p,0_*. The calculated *d_mod_* values are greater than *d_nom_* values for all modulator block sizes at 7 MeV, close to *d_nom_* values at 13 MeV, and less than *d_nom_* values at 20 MeV. The same trends are observed for *d_nom_* values of 0.223 and 0.315 cm blocks, not part of the measurements and fits, giving confidence that the quadratic fit using Equation (12) is appropriate. Fit parameters for Equation (12) are listed in Table 2.

**TABLE 2:** Fitted coefficients of Equation (12) quadratic function for the parameterization of intensity‐modulated PBRA diameter corrections

Coefficient	Value	Units
A_0_	1.61E‐02	cm
A_1_	1.09E+00	–
A_2_	−2.51E‐03	cm MeV^−1^
A_3_	−1.45E‐01	cm^−1^
A_4_	−1.40E‐03	MeV^−1^
A_5_	6.42E‐05	cm MeV^−2^

The improvements resulting from the modified diameters using Equation (12) are revealed in Table 3, which compares two metrics: (1) root‐mean‐square (RMS) comparing PBRA‐calculated with measured dose differences for the modulated (fitted) region and (2) passing rates for 2% dose difference or 2 mm distance to agreement. Results show significant improvement in RMS values and passing rates at 7 and 20 MeV and little improvement at 13 MeV due to the small calculated corrections in block diameter. Passing rates are 98–100% except for the 0.273 cm *d_nom_* value at 20 MeV and the 0.352 cm *d_nom_* value at 13 and 20 MeV. Not plotted is the good correlation between passing rates (%) and RMS (%).

**TABLE 3:** Accuracy of calculations performed with physical and optimized, modified island block diameters

	Island block diameter (cm)			Intensity reduction factor			2%/2 mm Passing rate (%)		RMS (%)	
Energy (MeV)	*d_nom_*	*d_IS_*	*d_mod_*	IRF*_nom_*	IRF*_IS_*	IRF*_mod_*	Nominal	Modified	Nominal	Modified
7	0.158	0.188	0.167	0.937	0.911	0.930	100.0	100.0	0.779	0.337
	0.273	0.306	0.283	0.812	0.764	0.797	98.6	100.0	1.521	0.348
	0.352	0.386	0.361	0.688	0.625	0.671	80.0	100.0	1.717	0.423
13	0.158	0.178	0.159	0.937	0.920	0.935	100.0	100.0	0.465	0.449
	0.273	0.294	0.274	0.812	0.782	0.811	100.0	100.0	0.885	0.883
	0.352	0.373	0.352	0.688	0.649	0.690	93.5	93.5	1.205	1.205
20	0.158	0.171	0.155	0.937	0.926	0.940	98.2	99.8	0.832	0.773
	0.273	0.286	0.269	0.812	0.793	0.817	79.0	85.4	1.626	1.515
	0.352	0.366	0.345	0.688	0.663	0.699	69.2	71.5	2.214	1.855

The modified island block diameters (d_mod_) and their intensity reduction factors (IRF_mod_) are compared with physical (nominal) island block diameters (d_nom_) and their IRF_nom_ values for the combinations of the three island block diameters and 7, 13, and 20 MeV beams. The in‐scatter adjusted diameters (d_IS_) and corresponding IRF_IS_ values, determined considering only in‐scatter effects, are listed for comparison. The accuracy of each calculation is evaluated by comparing its 2%/2 mm passing rate, which correlates well with the RMS (%) of the d_mod_ optimization within the modulated region where least squares was minimized

Table 3 also illustrates the dependence of *d_mod_* values on scatter into and from the island blocks. It compares *d_mod_* with *d_IS_* values as a function of *d_nom_* and *E_p,0_*, where *d_IS_* is the island block diameter predicted by the in‐scatter calculation using pencil beam theory (Equation 9). For all beam energies and block diameters, calculated *d_IS_* values increased relative to *d_nom_*, due to in‐scatter removing electron fluence from the beam. The magnitude of this change decreases with beam energy, as $\sigma_{\theta_{x},clinical}$ decreases. For the nominal 0.352 cm block, *d_IS_* decreases from 0.386 cm at 7 MeV to 0.373 at 13 MeV and 0.366 cm at 20 MeV. Calculated *d_mod_* values decreased relative to *d_IS_* for all beam energies and block diameters due to the incorporation of out‐scatter. At 7 MeV, *d_mod_* exceeds *d_nom_* as in‐scatter predominates. At 13 MeV, the in‐scatter and out‐scatter offset each other, resulting in *d_mod_* being approximately equal to *d_nom_* for all block sizes. At 20 MeV, *d_mod_* is less than *d_nom_* as out‐scatter predominates.

### 4.2 Comparison of PBRA calculations using modified island block diameters with measurement

**FIGURE 4:**
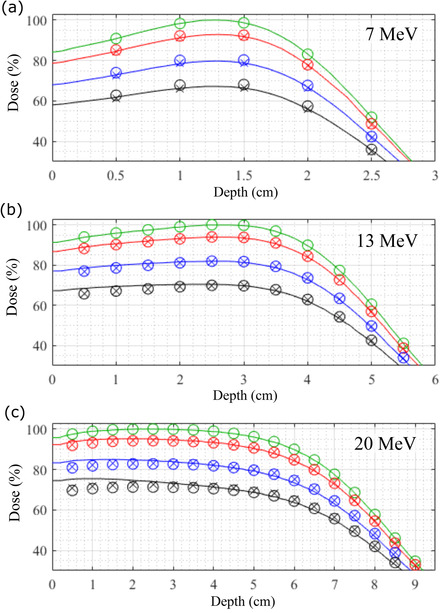
Comparison of PBRA‐calculated with measured central‐axis PDD curves for nominal and modified island block diameters. For the (a) 7, (b) 13, and (c) 20 MeV beams, the measured PDDs are plotted as solid curves, and PBRA‐calculated dose is plotted for the nominal (○) and modified (×) island block diameters. Results are normalized to *D_max_* for the foam only (no island blocks) setup, which are plotted in green. Results are plotted for PRIME devices having an 8.4×8.4 cm^2^ square (*cf*., Figure 2) containing island blocks with diameters of 0.158 (red), 0.273 (blue), and 0.352 (black) cm

This section compares measured with PBRA‐calculated dose distributions for both nominal (*d_nom_*) and optimized modified (*d_mod_*) island block diameters. These comparisons provide insight into where and why significant dose differences arise. Figure 4 compares measured with PBRA‐calculated central axis PDDs for the 7, 13, and 20 MeV beams for the four PRIME devices: foam only and 0.158, 0.273, and 0.353 cm diameter island blocks. The island blocks were positioned on a 0.6 cm hexagonal grid over an 8.4×8.4 cm^2^ area about central axis. PBRA calculations were made using both *d_nom_* and *d_mod_* values, the latter always agreeing better with measurement. The *d_mod_* results at 7 MeV agree well, within 1% for all depths and island block diameters. The *d_mod_* results at 20 MeV agree well, within approximately 1% for depths greater than 3 cm for all island block diameters. However, the magnitude of the calculation's underprediction of measured dose increases as depth decreases and island block diameters increase. For the 0.352, 0.273, and 0.158 cm diameter island blocks, dose differences are the greatest near the surface, being approximately 4.6%, 3.5%, and 2.4%, respectively, at 0.5 cm depth. The *d_mod_* results at 13 MeV are approximately midway between results at 7 and 20 MeV.

**FIGURE 5:**
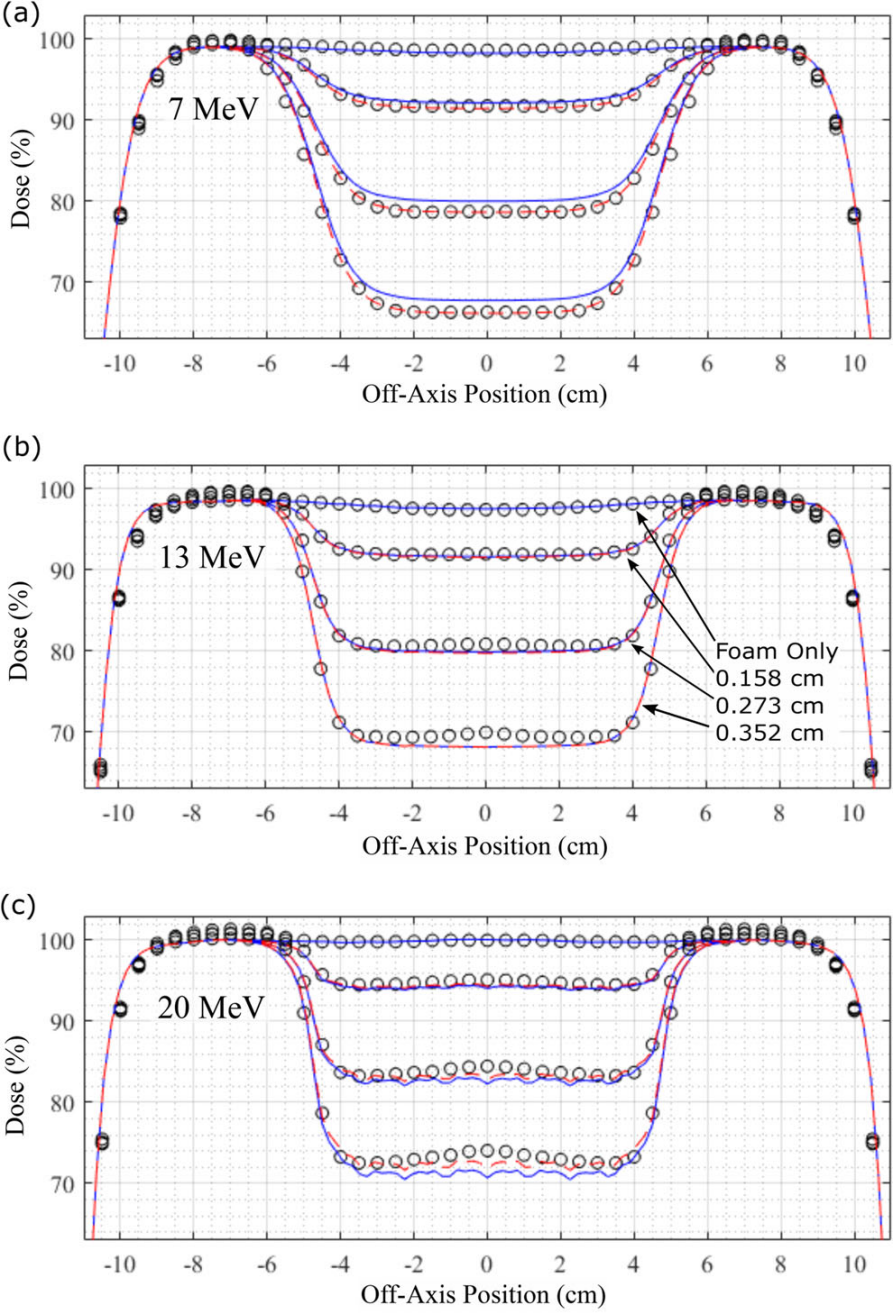
Comparison of measured and PBRA‐calculated profiles of dose versus off‐axis position beneath PRIME devices (*cf*., Figure 2). Profiles are compared for (a) 7, (b) 13, and (c) 20 MeV beams at depths in water of 1.0, 1.5, and 2.5 cm, respectively. Measured data (black circles) are compared with PBRA‐calculated dose using nominal island block diameters (solid blue curves) and modified island block diameters (dashed red curves). Percent dose values are normalized such that 100% = *D_max_* for the foam only setup, i.e. PRIME device with foam and no island blocks. Each plot shows comparisons for foam only and for island block diameters of 0.158, 0.273, and 0.352 cm, as indicated in (b)

**FIGURE 6:**
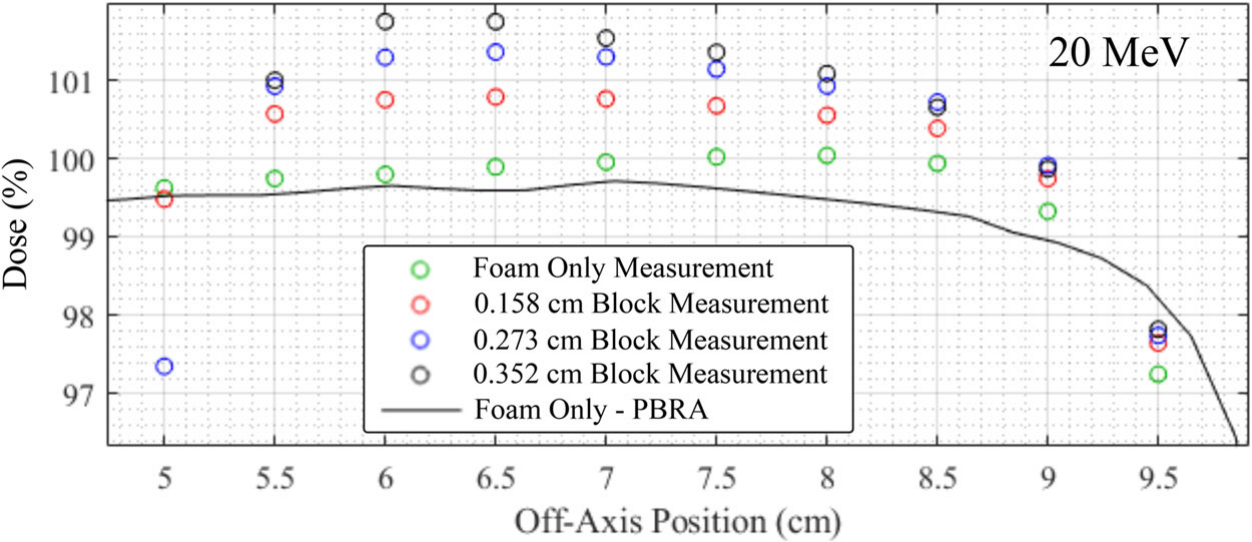
Comparison of off‐axis dose profiles in the unmodulated region of the 20 MeV beam for various island block diameters. The circles indicate profiles measured at 1.5 cm depth plotted verses off‐axis position for the foam only device (green) and for island block diameters of 0.158 (red), 0.273 (blue), and 0.352 cm (black). The solid black curve indicates the foam only PBRA calculation. Increasing dose values with increasing island block diameter is due to electron large angle scatter from the island blocks of the PRIME devices, which were restricted to central 8.4×8.4 cm^2^ square (*cf*. Figure 2)

Although dose differences between PBRA‐calculated and measured dose can be significant on central axis, particularly at shallower depths, higher energies, and larger island block diameters, these differences decrease away from central axis, as illustrated in Figure 5. This figure compares measured dose with PBRA‐calculated dose for both *d_nom_* and *d_mod_* values for foam only and 0.158, 0.273, and 0.352 cm diameter island blocks at depths of 1.0, 1.5, and 2.5 cm for 7, 13, and 20 MeV beams, respectively. In all cases, the *d_mod_* value gives the closest agreement, and the agreement improves as off‐axis distance increases. At 7 MeV the PBRA calculation using *d_mod_* agrees within 1% for all island block sizes inside the field's penumbra, just as was the case for central‐axis dose. At 20 MeV the PBRA calculations using *d_mod_* produce small dose differences for points further off‐axis, but remain significant near central axis, particularly for the larger island block diameters. Even in the unmodulated region outside the projection of the intensity modulators, the measured dose is approximately 2% greater than PBRA‐calculated dose, illustrating the far‐reaching extent of the block scattered electrons. This effect is illustrated in Figure 6, which magnifies the 20 MeV results at 5 to 9 cm off‐axis and 1.5 cm depth, showing measured dose to be as much as 2% greater for the 0.352 cm diameter measurements. At 13 MeV, the agreement between PBRA‐calculated and measured dose is between that at 7 and 20 MeV.

### 4.3 Evaluation of clinical utility of PBRA‐calculated dose distributions using modified island block diameter

**FIGURE 7:**
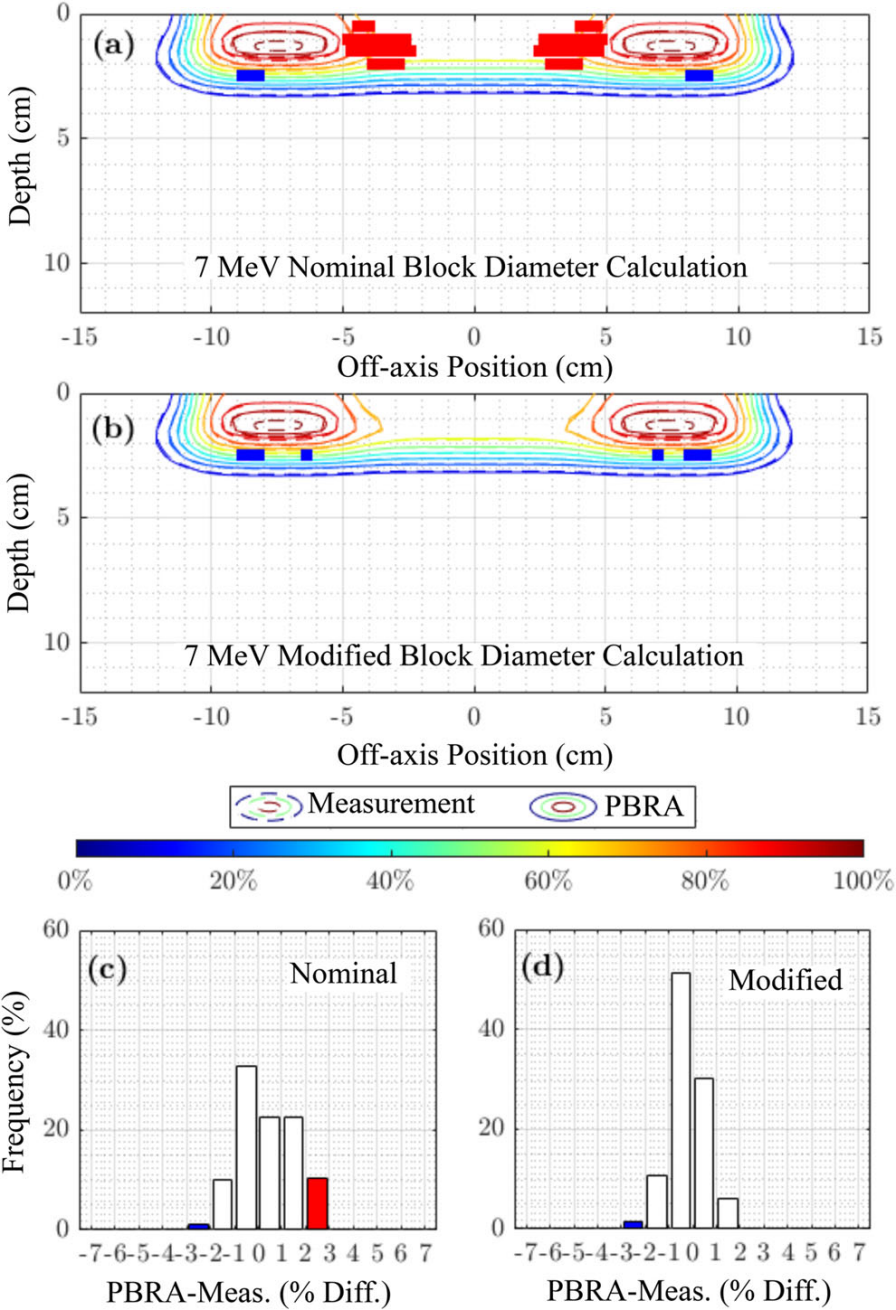
7 MeV comparison of PBRA‐calculated and measured, in‐plane dose distributions under PRIME device with 0.352 cm diameter (*d_nom_*) island blocks (IRF*_nom_* = 0.688). Measured isodose contours (solid curves), plotted every 10% from 10% (dark blue) to 100% (dark red), are compared with PBRA‐calculated isodose contours (dashed curves) calculated with (a) nominal and (b) modified island block diameters. 100% = central‐axis *D_max_* for the foam only PRIME device (without island blocks). Measured areas outside a 2%/2 mm passing criteria are indicated in blue (PBRA < measured dose) and red (PBRA > measured dose). Histograms of dose difference (PBRA calculated less measured dose) at the measured depths are plotted for (c) physical (*d_nom_*) and (d) modified (*d_mod_*) island block diameters. Values exceeding the 2%/2 mm passing criteria are indicated in blue and red, as in (a) and (b)

**FIGURE 8:**
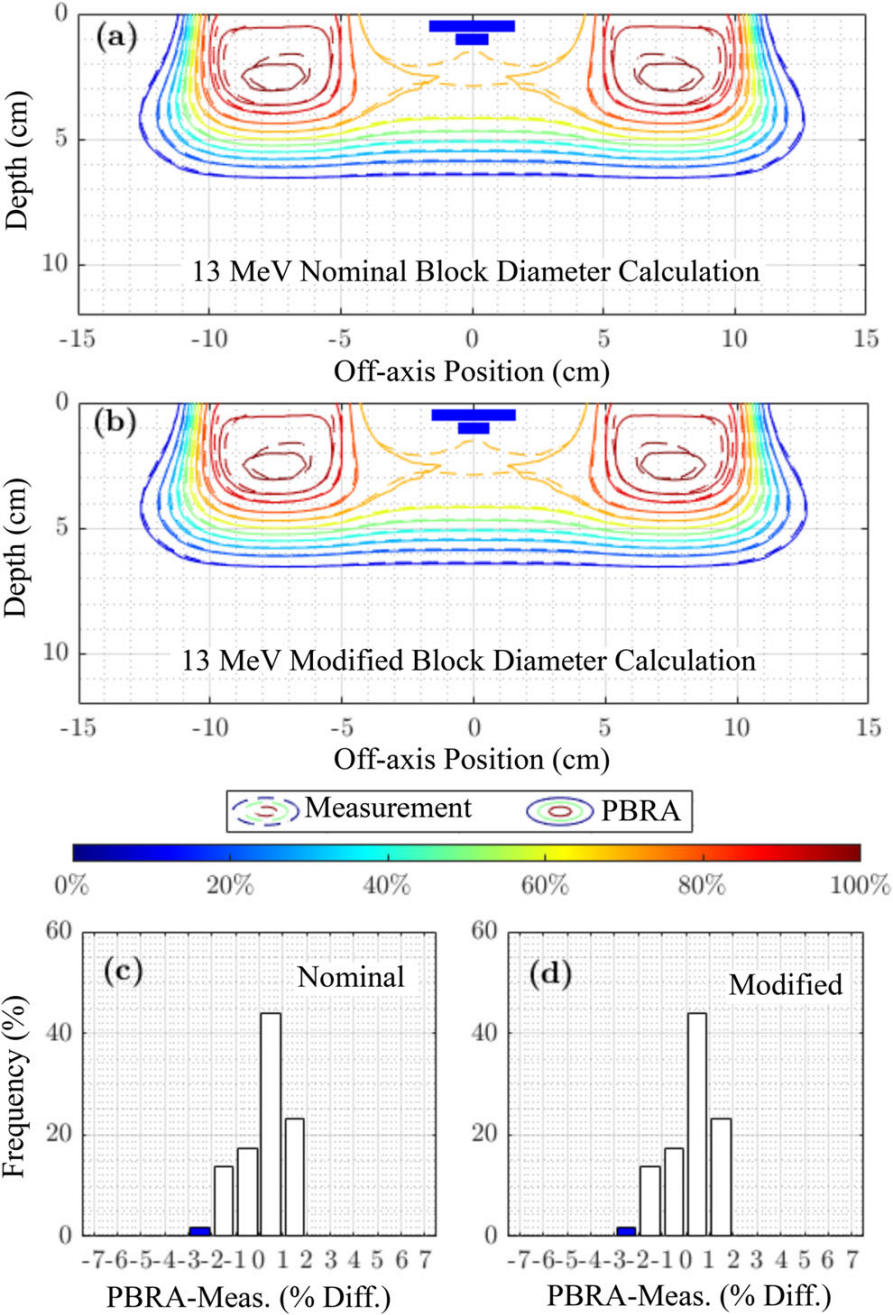
13 MeV comparison of PBRA‐calculated and measured, in‐plane dose distributions under PRIME device with 0.352 cm diameter (*d_nom_*) island blocks (IRF*_nom_* = 0.688). Measured isodose contours (solid curves), plotted every 10% from 10% (dark blue) to 100% (dark red), are compared with PBRA‐calculated isodose contours (dashed curves) calculated with (a) nominal and (b) modified island block diameters. 100% = central‐axis *D_max_* for the foam only PRIME device (without island blocks). Measured areas outside a 2%/2 mm passing criteria are indicated in blue (PBRA < measured dose) and red (PBRA > measured dose). Histograms of dose difference (PBRA calculated less measured dose) at the measured depths are plotted for (c) physical (*d_nom_*) and (d) modified (*d_mod_*) island block diameters. Values exceeding the 2%/2 mm passing criteria are indicated in blue and red, as in (a) and (b)

**FIGURE 9:**
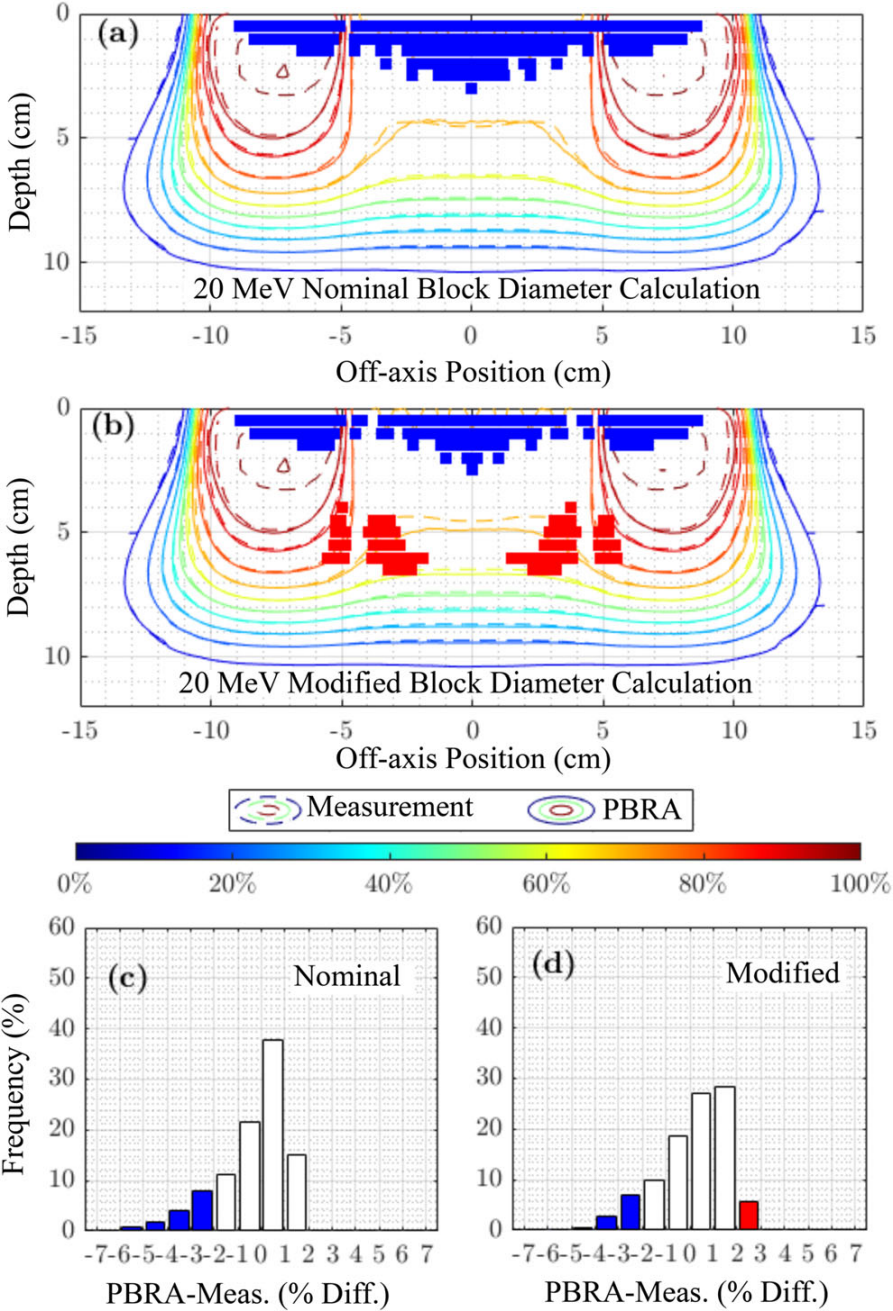
20 MeV comparison of PBRA‐calculated, and measured in‐plane dose distributions under PRIME device with 0.352 cm diameter (*d_nom_*) island blocks (IRF*_nom_* = 0.688). Measured isodose contours (solid curves), plotted every 10% from 10% (dark blue) to 100% (dark red), are compared with PBRA‐calculated isodose contours (dashed curves) calculated with (a) nominal and (b) modified island block diameters. 100% = central‐axis *D_max_* for the foam only PRIME device (without island blocks). Measured areas outside a 2%/2 mm passing criteria are indicated in blue (PBRA < measured dose) and red (PBRA > measured dose). Histograms of dose difference (PBRA calculated less measured dose) at the measured depths are plotted for (c) physical (*d_nom_*) and (d) modified (*d_mod_*) island block diameters. Values exceeding the 2%/2 mm passing criteria are indicated in blue and red, as in (a) and (b)

This section shows the overall accuracy of the nominal and modified PBRA‐calculated dose by comparing with measured planar dose distributions and examining pass rates for dose within 2% or 2 mm. For beam energies of 7, 13, and 20 MeV, Figures 7, 8, and 9, respectively, compare PBRA‐calculated isodose distributions for the largest island block diameter (*d_nom_* = 0.352 cm) against measured dose. Not illustrated in the earlier dose comparisons, agreement in the penumbral and distal falloff regions is excellent, within 0.1 cm. Calculation points where dose differences exceed 2% and 2 mm are labeled red if PBRA‐calculated dose is greater than measurement and blue if PBRA‐calculated dose is lesser.

The PBRA calculation using the *d_mod_* value at 7 MeV improves agreement over that using *d_nom_*; Table 3 shows improvement from 80.0% to 100% for the 0.352 cm island block. For the 0.273 and 0.158 cm block diameters, little improvement in passing rate was available, i.e., 98.6% to 100.0% for the former and both 100% for the latter. At 13 MeV, there was minimal change from *d_nom_* to *d_mod_*, resulting in no improvement to the passing rates, which were 100%, 100%, and 93.5% for the 0.158, 0.273, and 0.352 cm island block diameters, respectively. At 20 MeV, the reduced *d_mod_* value resulted in the passing rate for the 0.352 cm diameter island block increasing from 69.2% to 71.5%, and the isodose plots showed the large blue region being now more equally split between undercalculation (blue) and overcalculation (red). Passing rates for the 0.273 cm diameter island block showed most significant improvement, increasing from 79.0% to 85.4%, while the 0.158 cm diameter block passing rate increased from 98.2% to 99.8%.

## 5 DISCUSSION OF RESULTS

### 5.1 Impact of scatter on modified island block diameter

In‐scattered electrons, those scattered into the sides of the blocks, increased the effective diameter of the island blocks as a function of its physical diameter and beam energy; this results in a decreased IRF, as computed by Equation (1). This is illustrated in Table 3 for the three island block diameters and three beam energies selected for this study. At 7 MeV, the decreases in IRF values ranged from 0.026 for the smallest island block (*d_nom_* = 0.158 cm; IRF*_nom_* = 0.937) to 0.063 for the largest island block (*d_nom_* = 0.352 cm; IRF*_nom_* = 0.688). At 20 MeV, the decreases in IRF values range from 0.011 to 0.025, less than the decreases at 7 MeV. This is attributed to the scattering powers in air and the intensity modulator's machinable foam decreasing with increasing energy.

Out‐scattered electrons, those scattered out the sides of the island block, decrease the effective diameter of the island blocks as a function of its physical diameter and beam energy; this results in an increased IRF, as approximated by the optimization procedure and results presented earlier. Again, this is illustrated in Table 3 for the three block diameters and three beam energies selected for this study. At 7 MeV the increases in IRF values from those computed considering only in‐scattered electrons, ranged from 0.019 for the smallest island block to 0.036 for the largest. Similar increases are seen at 20 MeV, where the increases in IRF values from those computed considering only in‐scattered electrons, ranged from 0.014 to 0.036. This is attributed to the decrease in scattering power with energy being offset by the increased range of electrons in the island block.

Interestingly, because the in‐scattered and out‐scattered electrons counteract each other's effect, the resulting IRFs for the corrected island block diameters are within 2% of values based on the physical (nominal) diameters. At 13 MeV, the IRF values agree within 0.002 for all three island block diameters. Contrastingly, at 7 MeV the corrected IRF values are lower and within 0.017, whereas at 20 MeV, the corrected values are higher and within 0.011.

### 5.2 Utilization of effective island block diameters in electron intensity modulators

Increases and decreases of IRF values, that is, modified IRF values, result in corresponding decreases and increases in the corrected block diameters. The corrected block diameters are used for dose calculations using the intensity modulated PBRA dose algorithm, as explained earlier. Results showed that dose calculations using these corrected diameters should be sufficiently accurate for IM‐BECT considering the following. First, it is unlikely that an IRF value much less than 0.80 will be required for IM‐BECT. The patient head plan reported by Kudchadker et al.^4^ shows a significant gradient in the bolus surface that is unlikely to be exceeded in most clinical cases, and its minimum IRF is approximately 0.80. Results for the 20 MeV beam and 0.273 cm diameter island block (IRF*_nom_* = 0.812) showed the accuracy of dose calculation in the modulated region to be ± 3% for almost all points. The region where measured dose overestimated calculated dose is near the surface, and likely often falling inside the bolus. Additionally, this maximum error in dose calculation is at the center of an 8.4×8.4 cm^2^ grid of island blocks, an extreme geometry that is unlikely to be encountered in the clinic.

### 5.3 Recommendation for increasing accuracy of PBRA calculation

The current model of simply using a corrected diameter for each island block as a function of beam energy and diameter seems adequate for IM‐BECT and the range of conditions studied; however, there might be future applications of island blocks requiring smaller IRFs (larger island block diameters), different spacing (≠ 0.6 cm), or greater SSDs, in which case the current approximations might not be clinically sufficient. For such cases, it is recommended that an alternative method with better physical modeling be developed.

**FIGURE 10:**
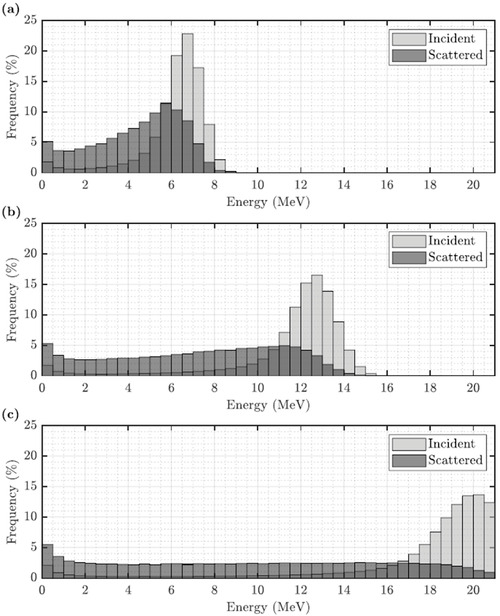
Comparisons of incident and island block‐scattered electron energy distributions. MC‐calculated histograms compare energy distributions of incident electrons (light gray) and island block‐scattered electrons (dark gray) for (a) 7, (b) 13, and (c) 20 MeV beams. Phase spaces were scored at 97 cm SSD for a 0.315 cm diameter, 0.6 cm long tungsten island block located 5 cm above isocenter (95 cm SCD)

The PBRA is structured in a way that should allow the following modeling. First, in‐scattered electrons can be modeled by the corrected diameter using the pencil beam model presented in the Theory section. Second, out‐scattered electrons can be modeled as a pencil beam at the location of each island block. It would be specified by the fraction of electrons incident on the island block that out‐scatter, their energy distribution, and their angular distribution. These distributions can be determined as a function of beam energy and block diameter using Monte Carlo. Examples of preliminary data from Scotto^28^ are illustrated in Figure 10. Once these phase space distributions are available, electrons can be propagated from the plane of the collimating insert to centers of pencil beams that first propagate through the patient surface. That propagation uses the same transport code that the PBRA currently uses to propagate electrons passing through the cutout to the patient's surface.^2^ Then, dose in the patient from the out‐scattered electrons could be calculated by an independent PBRA calculation that could be added to the PBRA dose calculation for the primary electrons, less those scattered into the sides of the island blocks.

## 6 CONCLUSION

The overall objective of this study was to achieve 2% dose accuracy or 2 mm distance‐to‐agreement for PBRA‐calculated dose distributions in a water phantom underlying passive intensity modulators (PRIME devices) with island block diameters in the range useful for IM‐BECT. A theoretical expression was derived that modeled scatter into the side of cylindrical island blocks. However, we were unable to derive such a model for electrons scattering from the side of the cylindrical island blocks into the beam. Therefore, the present work pursued an empirical model believed potentially sufficiently accurate for IM‐BECT use in the clinic.

Our empirical model, which slightly adjusted nominal island block diameters as a function of beam energy and nominal island block diameter to optimally agree with measured data under intensity modulators (least square fit in modulated portion of treatment volume), was unable to achieve our accuracy goal of 2%/2 mm at all beam energies studied. Although such agreement was achieved for all island block diameters studied at 7 MeV and for the smaller island blocks at 13 MeV, it failed increasingly for increasing island block diameters and beam energies, through 20 MeV and 0.352 cm island block diameter. The largest dose differences (4.6%) were on central axis at a shallow depth (0.5 cm) for the 20 MeV beam. The magnitude of dose differences became less at points further from central axis, deeper depths, lesser beam energy, and smaller island block diameter.

The 2% dose difference was achieved at depths beyond 2 cm at 20 MeV and 1 cm at 13 MeV, and there is high likelihood that these depths will comprise most of an IM‐BECT patient dose distribution. Also, these dose comparisons were made for a highly modulated PRIME device with island blocks placed every 0.6 cm on a hexagonal grid. Therefore, we hypothesize that our IM‐BECT PBRA calculations will achieve our 2% accuracy goal for actual patient intensity modulators. Such results will be evaluated in the future,^9^ as clinical quality assurance measurements are made for IM‐BECT PRIME devices.

The advantage of our empirical method of accounting for in‐scatter and out‐scatter is that it adds insignificant time of calculation to the PBRA dose calculation. However, if greater accuracy is determined to be needed in the future, this study computed preliminary MC data showing a continuous energy spectrum of out‐scattered electrons from a 0.315 cm diameter tungsten island block at 7, 13, and 20 MeV. For additional combinations of the multiple beam energies and island block diameters to be used clinically, MC could compute and store energy spectra and angular distributions. Such phase space data could be used for a supplementary PBRA calculation that would transport the out‐scattered electrons from the intensity modulator's island blocks through the patient. That dose distribution could then be added to the PBRA‐calculated dose distribution described in this study using only the island block diameter correction for in‐scattered electrons computed using the theory above. Such a method should easily meet the 2%/2 mm passing criteria, but at the expense of approximately doubling the time of PBRA calculation. Such an approach could be beneficial for uses of PRIME devices other than for IM‐BECT, that is, that require IRF values significantly less than those of 0.671 or greater in the present study.

## AUTHOR CONTRIBUTIONS

As submitting author, I attest that all coauthors and myself (1) contributed to writing and/or reviewing the submitted manuscript, (2) approved the final submitted manuscript, and (3) are accountable for the integrity of the material submitted. Each individual contributed in multiple ways to the material reported in this manuscript, and the primary contribution(s) from each author are:

- Joseph G. Scotto was a graduate student whose MS thesis comprised the bulk of the research for this project, particularly initial data measurements and all PBRA dose calculations and data analysis.
- Garrett M. Pitcher supervised both graduate students and shared manuscript writing.
- Robert L. Carver assisted the graduate student in the PBRA calculations.
- Kevin J. Erhart provided the PRIME devices used in this study, supervised coding of the treatment planning software used in the study, and served as principal investigator on the SBIR grant funding this study.
- Andrew S. McGuffey assisted with the final dose measurements reported in this study.
- Kenneth R. Hogstrom conceived the concept of intensity modulated bolus electron conformal therapy (IM‐BECT), developed the scientific portion of the grant that funded the project, contributed to data analysis, and shared manuscript writing.

## CONFLICT OF INTERESTS

Kevin Erhart is an employee of .decimal, LLC and principal investigator of NIH Award Number R41CA199838 for which Mary Bird Perkins Cancer Center has a subaward. The authors have no other relevant conflicts of interest to disclose.
